# Epidemiological analysis of athlete injuries in Muay Thai in-ring matches

**DOI:** 10.1186/s40621-025-00569-x

**Published:** 2025-05-26

**Authors:** Hasan Hallaçeli, Serkan Davut, Ali Özbek, Erdoğan Aydın

**Affiliations:** 1 Department of Orthopedics and Traumatology, Hatay Mustafa Kemal University, Hatay, Turkey; 2 Institute of Health Sciences, Hatay Mustafa Kemal University, Hatay, Turkey; 3 Private Lokman Hekim Esnaf Hospital MD. International referee, Head of IFMA’s Medical Commission, Aydın, Turkey

**Keywords:** Muay Thai, Athletic injuries, Sports

## Abstract

**Background:**

Compared to other sports, combat sports are typically thought to be dangerous and more prone to injury. The injury rates sustained by Muay Thai combat athletes during practice, competition, and tournaments are presented as an overall rate in the literature. However, none of the earlier studies have focused on injuries during official championship matches. Head trauma (5–66%), epistaxis, laceration, contusion (2–28%), soft tissue of the extremities (9–77%), and other conditions have been linked to high rates of disability. In addition to its distinct qualities and growing appeal, Muay Thai is an Olympic Committee and a UNESCO-recognized sport. Our primary goal was to assess the patterns, frequency, and severity of in-ring injuries and collect data for the sports authorities and the literature. The second goal was to determine the relationship between the “referee stop contest/RSC decision” and injury incidence.

**Methods:**

The ringside doctor examined all healthy athletes twice before and after the match. The number of athletes assessed from a combat perspective, the overall number of contests, and the percentage of athletes who maintained their health during the competition were ascertained. Furthermore, following the announcement of the RSC decisions, medical diagnosis, first aid, and hospital referral status were disclosed along with the decision rates.

**Results:**

This study included 663 athletes (445 males, and 218 women). A total of 606 athletes (91.4%) had no health issues following official bouts. It was observed that 24.58% of the contests were completed with the RSC decision. 68 athletes (10.25%) received medical treatment; 57 (8.60%) had their matches stopped by the RSC decision. The remaining 11 (1.65%) completed the competition without an RSC decision but requested a medical examination after the bout. According to our study, injuries related to the head and extremities were surprisingly low, at a rate of approximately 4%. Most of the patients required outpatient treatment. Epistaxis, concussion, rib trauma and extremity soft tissue strains were among the most frequent injury categories with percentages of 1.96%, 1.50%, 1.05%, and 1.05%, respectively. For the 17 individuals, the hospital emergency room attendance rate was 2.56%. An urgent operation was scheduled for one patient (0.15%) at the hospital.

**Conclusions:**

Compared to published research, injury rates are comparatively low in Muay Thai in-ring official contests managed by doctors and referees. The data we obtained suggest that RSC decision may be useful in preventing athlete injury.

## Background

Muay Thai, or Thai boxing, is a type of combat sport similar to jiu-jitsu, kickboxing, and mixed martial arts (MMA) [1–3]. Muay Thai has unique techniques and disciplines that differ from others. It, apart from kickboxing, allows hard blows to the opponent’s skull from knee to elbow, so this anatomical region has attracted the attention of the authors [4–6]. Combat sports are generally regarded as more dangerous and injury-prone than other sports because of striking, throwing, or immobilizing an opponent [4, 6]. According to the Muay Thai tradition, athletes in official matches are expected to hit each other hard on all parts of the body, except the genitals, in three rounds of three minutes each [5, 7]. An athlete can exert about 1850 Newtons of force on the opponent’s thigh during a Muay Thai match by using the low-kick technique [8]. The referee’s directive is to give the boxer with the best style and defense the win [7]. The overall injury rates of Muay Thai martial athletes during practice and competition have been documented in previous research [6, 9–12]. For many anatomical locations, high rates of problems were highlighted, including head trauma (5–66%), epistaxis, laceration, and contusion [4, 12, 13]. 2–28% [14–16]; soft tissue issues in the extremities 9–77% [9, 17]; and maxillofacial issues 7–69% [16, 18, 19]. Muay Thai’s rapid popularity has resulted in an estimated two million participants worldwide [2, 5]. Surprisingly, UNESCO has acknowledged the uniqueness of Muay Thai sports, particularly in terms of positive youth development. In addition, the International Olympic Committee [20] recognized the Muay Thai Federation [5] in 2016.

Strict guidelines have been set in place by the International Federation of Muay Thai Associations (IFMA) to safeguard the “health and safety of athletes” and guarantee competitive, fair sports [5]. These are age limit for athletes, weight in, athlete equipment (head, shin guard, etc.), medical examination, doctor procedures, and decision of the referees: Referee Stops Contest / stopped match (RSC) [4, 5, 20]. The contest stops, according to the referee’s opinion (IFMA regulations), if the competitor is unable to continue because of physical limitations or injuries from legal strikes or other actions. Following the RSC decision, the referee has to follow the doctor’s advice on the ring. The competition shall be stopped if the referee decides an athlete is in danger, punished severely, or hit hard [5]. Establishing observation systems and analysing causal factors in homogenized environments is crucial for measuring the frequency and type of injuries [4]. Creating injury surveillance systems that gather precise injury data in the ring could be crucial for future championships, however, none of the earlier studies have examined injuries sustained during official championship matches.

Our primary goal was to assess, frequency, and severity of in-ring injuries and collect data for the sports authorities and the literature. The second goal was to determine the relationship between the “referee stop contest/RSC decision” and injury incidence.

##  Methods

### Participants

This study included 663 licensed athletes registered with the Turkish Muay Thai Federation. Our cohort included licensed female and male athletes (18–40 yrs) who competed in the February 2020 Seniors and the March 2022 Seniors Turkey Championships. Clothing and protective equipment for injury prevention: Genital organs, chest, trunk, head and shin protectors, mouth guards, hand bandages, and boxing gloves were used as mandatory. The World Health Organization’s (WHO) definition of health is that athlete performance health should be by the best physical, mental, and social well-being of an athlete related to sports success [21]. The universe of our study was formed by athletes who were found healthy according to the WHO definition by ring doctors and allowed to participate in the championships.

## Procedures and data collection

The Turkish Muay Thai Federation Medical Health Commission includes two doctors, one physiotherapist, one nurse, and ambulance team members. Athletes competition and fighting management were performed under standard conditions per the IFMA guidelines.

Before the competition, every athlete was examined by an approved doctor, and the Medical Declaration for IFMA competitors form, which contains the fighters’ age, gender, and weight data, was filled out. Throughout the championship, the authors and ring doctor were constantly present at the ringside. The authors recorded the round time and results of the contests with RSC decisions or not. Besides, medical data, including injuries causing RSC, medical diagnoses, and doctor treatment procedures for each athlete on the registration form in the presence of the ringside doctor, were noted. Information on the diagnosis and treatment of patients referred to the hospital was obtained and recorded from IFMA doctor reports.

## Research parameters

The ring doctor performed the first examination on all healthy athletes before the tournament. Each athlete was re-examined at the end of the bout. The diagnosis (according to the ICD 11 code) and relevant treatment of athletes injured during in-ring competition were noted in the athlete files and signed by the ring doctor. Information about the athletes’ health was categorized as follows:

A- Out of the ring and before the matchFinal checks of the athletes were made in the championship area. Competition athletes were divided according to sex, age, and weight categories (Table 1).

**Table 1:** Demographic characteristics of the athletes

Year/Championship	2020		2022		2020 and 2022	
Characteristics	***n***	**%**	**N**	**%**	**N**	**%**
n (number of fighters)	313	100	350	100	663	100
GENDER						
Male	205	65.5	240	68.5	445	67.1
Female	108	34.5	110	31.5	218	32.9
AGE						
18–23	270	86.2	290	82.8	560	84.4
25–40	43	13.8	60	17.2	103	15.6
WEIGHT CATEGORY						
Light (≤ 147 lbs.)	216	69	211	60.2	427	64.4
Heavy (> 147 lbs.)	97	31	139	39.8	236	35.6
n (number of bout)	266	100	325	100	591	100
BOUT /MATCHES (Sd:1.84)						
1–2 fights	257	81.4	267	76.2	524	79.0
3–4 fights	54	17.2	75	21.4	129	19.4
≥ 4 fights	2	1.4	8	2.4	10	1.5
Total fights	313	100	350	100	663	100

B- During and after the in-ring match:

At the end of the rounds, the athletes were divided into two groups “healthy” or “injuries”. If there were any indications of injury or a medical suspicion—which was regarded as an in-ring health issue—the match stopped under the referee’s direction. The athlete’s “healthy” or injured health information was recorded in their patient file along with the RSC decision. These:

a- Healthy, uneventful: Those without any injuries were noted as “healthy” in the championship success information.

b- RSC decisions for any time-in-ring match were recorded in the five subgroups. These.

1. RSC outclass: if the athlete was observed as incapacitated or unfit,
2. RSC-knock down: knocked down or unable to stand up quickly.
3. RSC-injury: any suspicious injury related to the extremity,
4. RSC-head: head-related injury and.
5. RSC-body: body-related injuries were reported in this study.

The first two are the decisions taken directly by the referee, while the others are the decisions taken by both the referee and ring doctor.

To conduct the analysis, the total number of matches and the proportion of athletes who remained healthy during the championship were determined. The RSC decision rates and medical diagnosis, first aid, and hospital referral status after the RSC decision were reported (Table 2).

**Table 2:** Stop contents ratios with a match-ending requirement

Year/Championship	2020		2022		2020 and 2022			
Number of fighters	313		350		663			
Sex	Female ( n/%)	Male ( n/%)	Female ( n/%)	Male ( n/%)	Female ( n/%)	Male ( n/%)		Total RSC
RSC: Referee Stops Contest								
I-RSC: According to referee (unfit condition): (n: 67)								
**Outclassed/Safety**	8 (2.55)	4 (1.27)	30 (8.57)	25 (7.14)	38 (5.73)	29 (4.37)		67 (10.10)
**Knockdown**	0	0	0	0	0	0		0
II-RSC: Doctor advises to stop the contest (n:96)								
**Injury (extremity)**	6 (1.91)	13 (4.15)	0 (0.00)	4 (1.14)	6 (0.90)	17 (2.56)		23 (3.46)
**Body**	4 (1.27)	11 (3.51)	6 (1.71)	11 (3.14)	10 (1.50)	22 (3.31)		32 (4.82)
**Head**	0 (0)	13 (4.15)	2 (0.57)	11 (3.14)	2 (0.30)	24 (3.61)		26 (3.92)
**Emergency to hospital**	3 (0.95)	6 (1.91)	2 (0.57)	4 (1.14)	5 (0.75)	10 (1.50)		15 (2.26)
**Total RSC**	21 (6.70)	47 (15.01)	40 (11.42)	55 (15.71)	61 (9.20)	102 (15.38)		163 (24.58)

According to IFMA guidelines, the referee announces the RSC decision as RSC outclassed (injuries suspected or unfit sports performance) or the presence of an RSC injury.

### Statistical analysis

IBM Statistics SPSS 20 for Windows (IBM Corp, Armonk, NY, USA) statistical software was used to analyse the data. Kolmogorov-Smirnov and visual (probability and histogram) graphs were used to assess the variables’ suitability to a normal distribution. The mean and standard deviation values were provided for the normally distributed variables, while percentages (%) were provided for the other variables. A variety of statistical analyses were conducted, including Pearson chi-square tests. 0.05 was regarded as a significant “p” value.

## Results

A total of 663 athletes—313 (205 men and 108 women) from the February 2020 Turkish Championship and 350 (235 men and 115 women) from the March 2022 Turkish Championship—were included in the study. There were 591 bouts (matches) at the National Championships, 266 in 2020, and 325 in 2022 (Table 1).

### RSC decisions

The athlete was declared ‘outclassed’ by the referee, and injuries to the head and body (extremities) were categorized by the ring doctor. Approximately 25% of competitor matches were found to have concluded with the RSC’s decision. The referees reported that 67 competitors were unfit, accounting for 10.10% of the total, while 96 athletes had injuries (suspected or not) in various anatomical parts, representing 14.47% of the total. In our investigation, it was found that 14 of every 100 fighters required medical evaluation and 2 of every 100 fighters required to transfer to emergency service (Table 2).

**Fig. 1:**
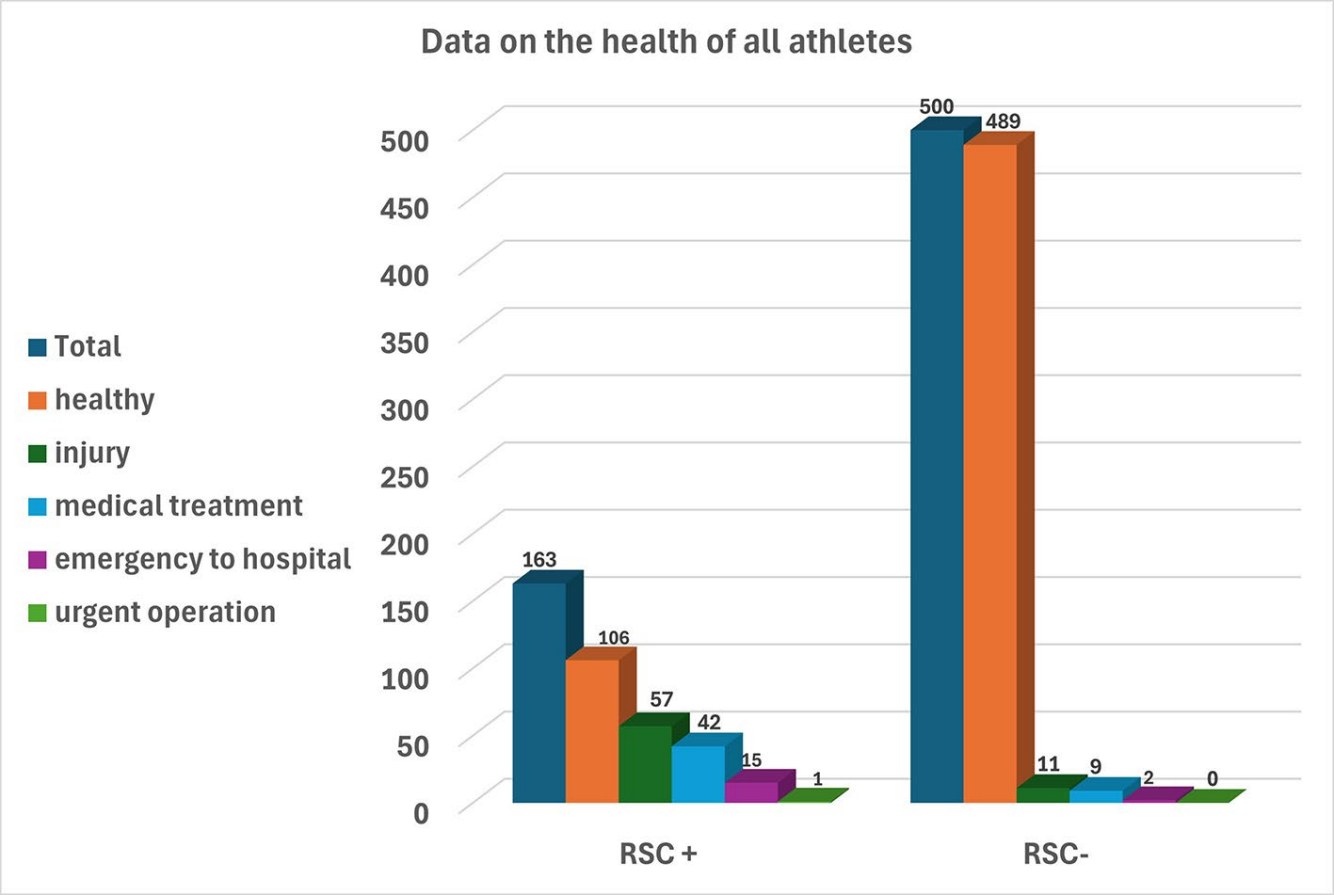
Injury occurred in 57 athletes with RSC(+); 42 athletes were treated at ringside, while 15 athletes were sent to the emergency room. Injuries were detected in 11 athletes with RSC (-); 9 athletes were treated at ringside, while 2 were sent to the emergency room. Only 1 athlete (RSC+) underwent urgent surgery

595 athletes (89.74%) did not experience any health problems, even painful cramps, ecchymosis, abrasion etc. (Fig. 1). The rate of being examined by the ring doctor as a result of the referee’s prediction or the injury observed in the athlete is 10,25% for 68 athletes (Table 3). 

**Table 3:** Athletes requiring ringside medical treatment and their diagnoses

National Championships							
**Year/ number of fighters**	**2020** **n:313**		**2022** **n:350**		**2020 and 2022** **n:663**		**ICD-11 Injury Code**
First Examination	n	%	n	%	n	%	
Syncope	2	0.63	2	0.57	4	0.60	MG45 Syncope and collapse
Concussion	4	1.27	6	1.71	10	1.50	NA07.0Z Concussion, unspecified
Laceration of eyebrow	7	2.23	4	1.14	11	1.65	NA01.2 Laceration of eyebrow
Jaw skin injury	1	0.31	2	0.57	3	0.45	NA01.Z Open wound of jaw
Lip injury	0	0	2	0.57	2	0.30	NA00.4 Superficial injury of lip or oral cavity
Epistaxis	9	2,87	4	1.14	13	1.96	MD20 Epistaxis
Rib trauma/pain	5	1,59	2	0.57	7	1.05	NB3Z Injuries to the thorax, unspecified
Acute abdominal trauma	1	0.31	0	0	1	0.15	NB91 Injury of intra-abdominal organs
extremity soft tissue strain (1st stage)	6	1.91	1	0.28	7	1.05	FB6Z Soft tissue disorders, unspecified
Knee ligament injury	3	0.95	0	0	3	0.45	NC93.Z Dislocation or strain or sprain of joints or ligaments of knee, unspecified
Shoulder dislocation	1	0.31	3	0.85	4	0.60	NC13.0 Dislocation of shoulder joint
Stomachache	0	0	2	0.57	2	0.30	MD81.10 Pain localized to upper abdomen
Fracture	0	0	1	0.28	1	0.15	NC53 Fracture at wrist or hand level
**Total injury**	**39**	**10.22**	**29**	**7.14**	**68**	**10.25**	

At the official Muay Thai contests, 68 athletes received medical care from the ring doctor

According to the ring doctor’s examination, the hospital emergency department referral rate for 17 individuals was 2.56%. Out of all hospital patients, just one (0.15%) received a recommendation for an urgent operation; 16 patients (2.41%) received recommendations for medication and rest (Table 4). Eleven (1.65%) athletes received medical advice following the match without an RSC decision (Fig. 1).

It was observed that 11 athletes (1.66%) who completed their matches regularly applied to the tournament physician with complaints of injuries after the match (Fig. 1).

After a more forceful fight in the ring, it was observed that there was a remarkable difference in the rates of hospitalization and injuries between those who received an RSC decision and those who did not (Table 5). Male athletes had a higher and statistically significant (*p* = 0.003) referral rate to emergency departments, even though both genders required equivalent health examinations (*p* > 1.45).

**Table 4:** Patients who were referred to the hospital emergency department and their diagnoses

Year/ Number of fighters	Championships in 2020 and 2022 (*n*:663)			RSC decision	ICD-11 Injury Code
Diagnosis	*n*	%	Treatment		
Knee ligament injury	1	0.15	Rest. PRICE protocol	-	NC93.Z Dislocation or strain or sprain of joints or ligaments of knee, unspecified
Suspicion of concussion	1	0.15	No medication	-	NA07.0Z Concussion, unspecified
Suspicion of concussion	4	0.60	No medication	+	NA07.0Z Concussion, unspecified
Jaw skin injury	1	0.15	Suturing	+	NA01.Z Open wound of jaw
Epistaxis	1	0.15	Tampon	+	MD20 Epistaxis
Rib trauma/pain	4	0.60	Rest. NSAID	+	NB3Z Injuries to the thorax, unspecified
Acute abdominal trauma	1	0.15	Urgent surgery	+	NB91 Injury of intra-abdominal organs
Knee medial collateral ligament injury	1	0.15	Straın 1/Rest. PRICE protocol	+	NC93.5 Strain or sprain involving fibular or tibial collateral ligament of knee
Shoulder dislocation	2	0.30	Closed reduction	+	NC13.0 Dislocation of shoulder joint
Phalanx fracture	1	0.15	Cast immobilization	+	NC53 Fracture at wrist or hand level
TOTAL	17	2.56	-		

Right after the bout, the ring doctor examined two athletes who didn't have an RSC decision

**Table 5:** Health status of athletes with and without RSC decision

Medical Treatment Requirement (Ringside)						
	Yes		No		X^2^	P
	n	(%)	n	(%)		
RSC Decision						
Yes	57	8.6	106	16.0	143.410	<0.001
No	11	1.7	489	73.8		

Rates of referral to hospital emergency departments according to RSC Decision						
Medical Treatment Requirement						
	Yes		No		X^2^	P
	n	(%)	n	(%)		
RSC Decision						
Yes (n:163)	15	2.3	148	22.3	38.124	<0.001
No (n:500)	2	0.4	498	75.1		

Rates of RSC Decision According to Weight						
	Weight					
	Light		Heavy		X^2^	P
	n	(%)	n	(%)		
RSC Decision						
Yes	79	11.9	84	12.7	3.281	0.07
No	283	42.7	217	32.7		

During the in-ring fight, 15 athletes were sent to the emergency room before the end of the 3rd round (with RSC), and 2 were sent after the end of the 3rd round (without RSC). (p) PearsonChi-Square test.

There is a minimal risk of being referred to an emergency hospital. Even in those who were not given an RSC decision, it was determined that there was an injury that required referral to the hospital, although very few (*p* < 0.001).

## Discussion

This study was the first prospective research to report injuries sustained during in-ring competitions at national official Muay Thai championships. 68 athletes (10.25%) received medical treatment; 57 (8.60%) had their matches stopped by the RSC decision. The remaining 11 (1.65%) completed the competition without an RSC decision but requested a medical examination after the bout with different complaints (Table 3).

In combat sports, concussion rates range from 5 to 66% [2, 6, 22, 23], while epistaxis rates range from 2 to 28% [14–16]. Some writers observed head area problems at a high rate of 66% in a small cohort of athletes from various combat disciplines [14, 15, 18]. It is understood that the survey method is preferred according to the statements of trainers and athletes in studies determining the epidemiology of injury [4, 12, 13, 22]. Surveys can efficiently gather large amounts of data from trainers and athletes about their experiences, perceptions, and the prevalence of injuries. However, some publications also state that, based on medical records, the correct response rate for an athlete’s self-identification of injury is 61% [13]. There is occasionally a greater percentage of self-diagnosis among athletes due to their trust in their ability to identify and evaluate their concussion symptoms. The 43.3% coach diagnosis rate, compared to the 79% athlete self-diagnosis rate for concussion reported in Follmer’s study, suggests that coaches may be unable to confirm such injuries through observation [12].

When the literature is reviewed, it becomes apparent that the injury rates of athletes in various disciplines such as Muay Thai, kickboxing, and MMA are reported retrospectively and cumulatively, regardless of whether they occur during training or contests [4, 6, 11, 12, 18]. In their retrospective analysis, Jensen et al. observed that 70–82% of injuries reported in MMA, Muay Thai, boxing, and taekwondo sports occurred during training, while 22–30% occurred during competitions [15]. MMA’s distinguishing trait is the ability to hit hard, choke, and lock the joints. There is also no necessity for protective equipment [15]. Rainey revealed that 77.9% of 55 MMA fighters in the United States were injured during training and 22.1% during competition. The most often injured region was the head/neck (38.2%), followed by the lower (30.4%) and upper (22.7%) limbs [23]. Shirani [18] reported a maxillofacial injury rate of 79% (*n* = 120) in various combat sports disciplines, whereas Chatrchaiwiwatana [24] reported 23.5% (*n* = 260). These include facial lacerations, bone fractures, dental injuries, and mandibular dislocations. According to Polmann [3], the overall pooled prevalence of dentofacial injuries in combat sports was approximately 30%. The most common of these are; wrestling, judo, boxing and jiu-jitsu branches are coming [3]. Our study determined that the rate of simple abrasions or bleeding in the facial area was unexpectedly low, approximately 4,37% for 663 athletes (Table 3).

**Fig. 2:**
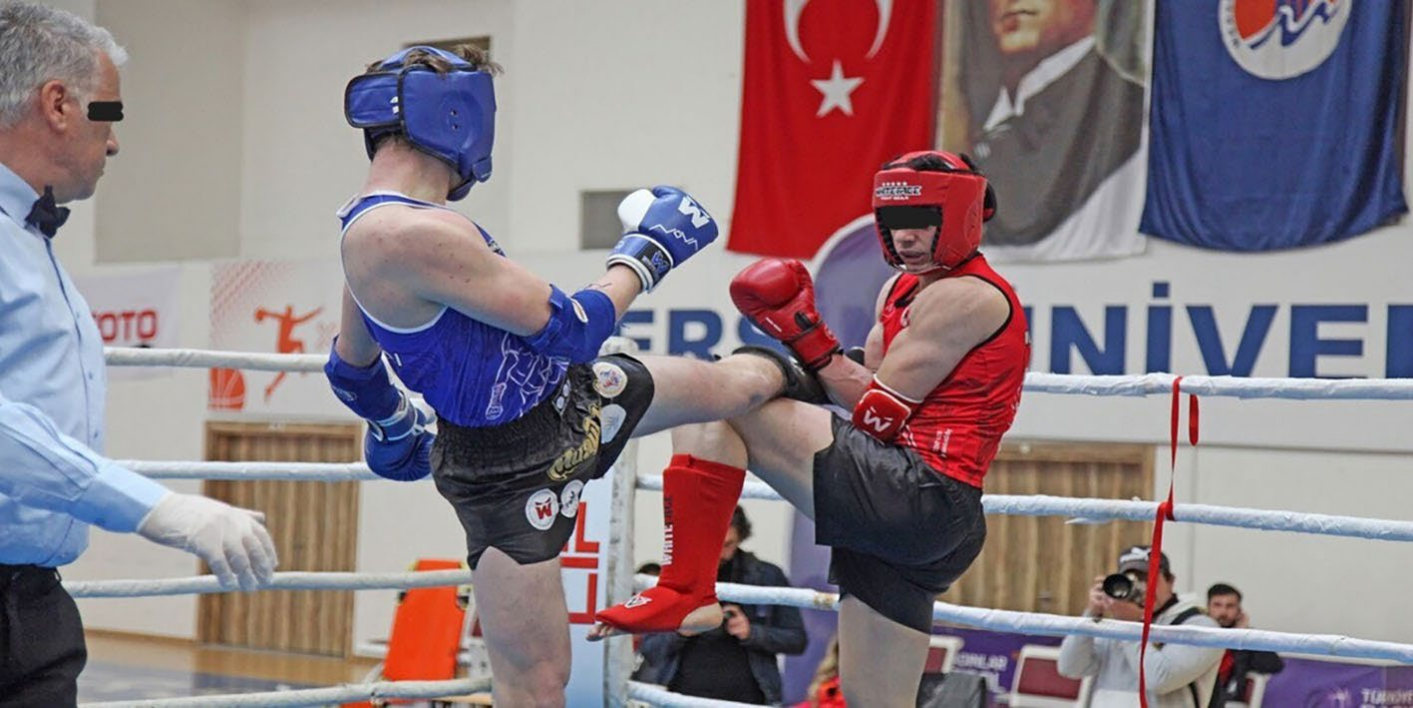
Defence or blocking technique of the opposite player during the legal strike to the body. The blue athlete’s action appears to be a legal kick, while the red athlete’s defensive maneuver—using his upper and lower extremities to block the kick—demonstrates a good technique to minimize the risk of injury. This situation highlights the importance of proper form and technique in contact sports, as well as the role of defensive strategies in ensuring athlete safety

Hill et al. [25] examined the maxillofacial injuries of 790 individuals who required emergency care, highlighting that they were most frequently associated with rugby (26.2%), cycling (23.9%), and football (16.4%). Surprisingly, the rate is relatively low among Muay Thai athletes, ranging between 0 and 3.2% [3, 9, 26]. According to Laoruengthana et al. [10], the majority of injuries occurred in rugby, handball, and basketball during the 2008 Thai National "Phitsanulok" Games (n=14,429 persons). Facial bone fractures that require surgery occur at an 8.1% rate in rugby, cycling, and football injuries [18]. To clarify, the increased occurrence of maxillofacial injuries in collision sports such as rugby, cycling, and football is related to players' vulnerability to direct or indirect strikes to the head or face, rather than in combat sports such as Muay Thai. While collision sports frequently rely on the unavoidable nature of contact, combat sports such as Muay Thai emphasize defensive abilities to manage and limit the risk of injury. Although Muay Thai allows legal strikes to the body by its nature, the opponent must be able to apply the defensive method (Fig. 2). Gartland investigated the frequency of injuries sustained during Muay Thai competitions, when protective equipment is mandatory, and reported that the rate increased as body weight decreased [14]. Pickering declared that not wearing mouth/dental protection during training is a health risk [16]. Muay Thai competitors who do not use protective equipment are more likely to suffer head injuries (5.4%), extremities injuries (55.4%), bruises/contusions (38%), lacerations, cuts, and swelling (27%), and fractures (12%) [4]. Injury rates vary because athletes do not have to use protective equipment during training or legitimate professional fighter conflicts, which are held for money and champion titles [4, 15, 18]. In the current study, amateur athletes' usage of mandatory protective equipment and the execution of appropriate stop-contest decisions explain the decreased injury rate compared to prior literature (Table 4).

According to Diesselhorst, joint sprains/muscle strains, abrasions/bruises, upper extremity dislocations, and fractures occur at rates of 47%, 26%, 47%, and 29% in MMA fighting, respectively. The prevalence of soft tissue disorders in the lower extremities ranges from 2 to 77% [9, 14–18]. In our study, injury rates in both the upper and lower extremities were lower (2%) compared to prior studies. Even though the athletes, who are strong by nature, fight each other, there is an aphorism of protecting the health of the athletes. Thus, the referee’s RSC decision is important. In official matches, if the athlete has difficulty executing an active movement or experiences extreme pain as a result of a legal blow, the RSC decision is applied immediately to protect the player’s health (Table 2).

Particularly in Muay Thai, referees’ RSC adjudications should be more explicit, regardless of players’ risk-taking expertise or capacity to regulate danger over time (6). The limited published data on Muay Thai and other sports is insufficient for comparison with this sport, currently being considered eligible for the Olympics. In the matches governed by the IFMA directive, injuries give less impression than the literature. Underestimating the risk of injury in training and the associated overestimation of the ability to negotiate risk may explain the high injury rate [6]. We think that the risk perceptions of the referees, ring doctors, and the players against injuries are different. This issue should be investigated in future studies.

**Fig. 3:**
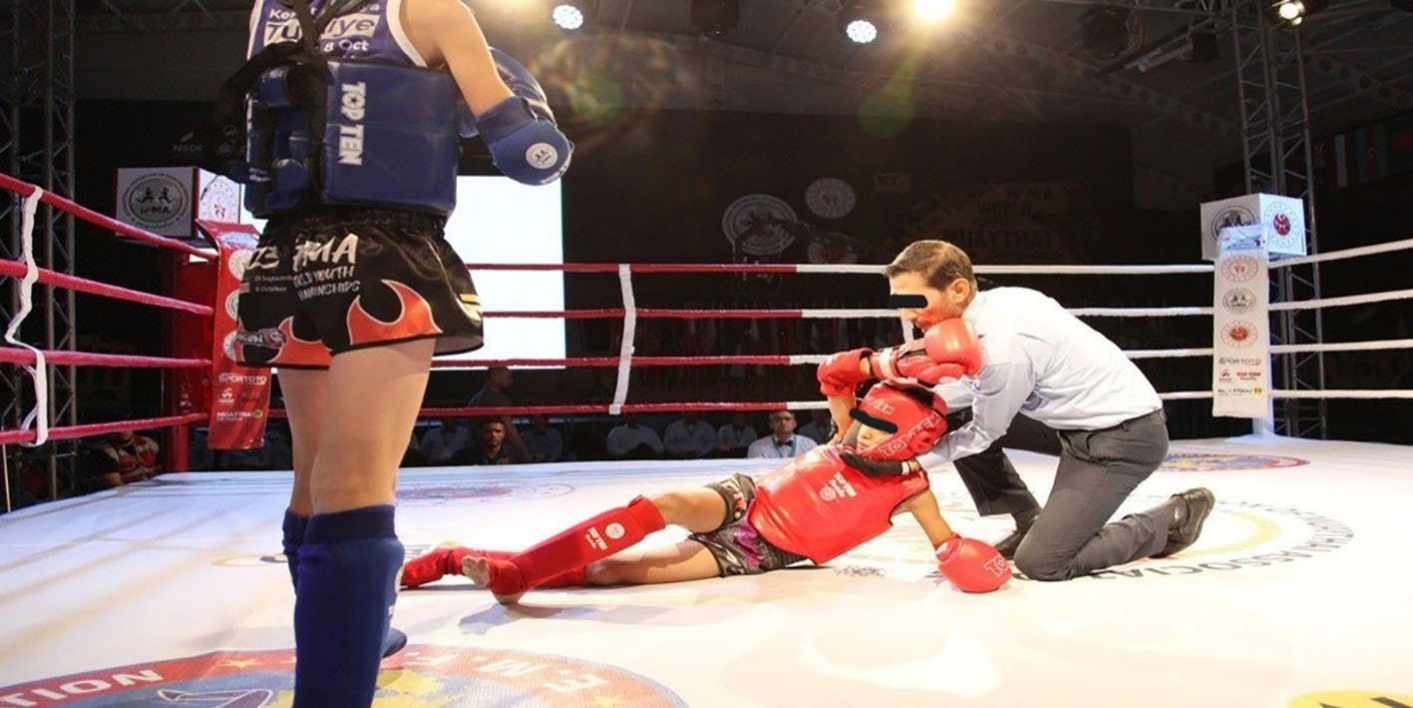
Any athlete who falls to the ground for any reason is protected by the Muay Thai referee. An RSC (Referee Stop Contest) decision is typically made when there is a concern for an athlete’s health and well-being, such as after a significant impact or fall. Adequate measures are often taken in sports to prevent injuries from both hard hits and falls

**Fig. 4:**
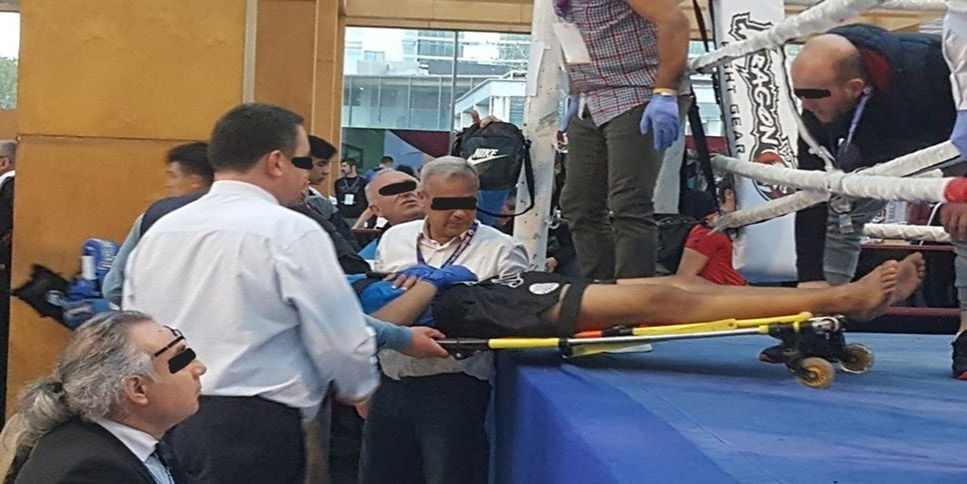
Ring doctor ends the match by RSC decision. The athlete was transported to the hospital emergency department and diagnosed with a mild strain in the medial collateral ligament of his right knee

It has been understood that in matches where the ring doctor is present, the referee is determined to finish the contest without any injuries to the athletes. Our findings indicate that the sensitivity of the RSC decision in predicting sports injuries is substantial in the Muay Thai discipline (Table 5). However, comparative studies with other sports are needed. The referee’s RSC decision is a critical announcement during the fighting where the referee decides to protect the health of the other athlete’s before announcing the win decision (Fig. 3). The RSC- outclassed decision may be taken if both competitors are healthy, and in exceptional situations. Even if there is no RSC decision, in exceptional situations, an athlete may be determined to have an injury that necessitates immediate transport to the hospital (Fig. 4). Participation in sports is not without risk. Strotmeyer noted that athletes who underestimate the risk of injury engage in more risk-taking behavior, while athletes who overestimate the risk adopt preventive behaviors [6]. Furthermore, some athletes may exaggerate their pain or anxiety to attempt the opponent’s powerful strikes. Our findings showed that just a small number of athletes with an RSC decision were later determined to be healthy by a medical professional. The physician concluded that there was no need for treatment (5.88%). Rarely do athletes who do not have an RSC decision require medical attention from a ringside physician (1.66%) (Fig. 1). However, Buse emphasized the significance of continuous medical supervision during the fight to guarantee that no severe medical repercussions occur in bouts performed under existing competition regulations [26].

No other study in the literature has presented data with this aspect. Records of health issues encountered during training and competition should be examined individually. In the matches managed with the coordination of the ring doctor and the IFMA directive, injury rates were found to be exceptionally low compared to the literature.

## Limitations

Professional Muay Thai official contests and Muay Thai training without protective gear are not included. Injuries sustained during training and formal competitions that are not supervised by a doctor or referee are not included. Analyses of fighting categories under 18 and over 40 were not possible. Athlete health-related psychological factors like competitive anxiety, etc., were not mentioned. Compared to the literature, this descriptive cross-sectional study found that there are fewer major injuries requiring hospital referrals, despite having a large sample size (*n* = 663 athletes). Therefore, based on statistical research, it was determined that the number of injuries is insufficient to demonstrate a cause-and-effect link. It is our belief that a healthy cause-and-effect relationship among injuries will be established with the collection of further data from future champions.

## Practical implications

Despite the similarities between sports injuries associated with fighting, each combat sport should be assessed independently.

It is important to examine amateur athletes’ injuries during training and competition separately.

Muay Thai athletes should be encouraged to use protective equipment throughout training.

In order to evaluate its impact on reducing the possible injury rate, it is crucial to gather information regarding sports-related injuries as well as the RSC decisions made by the referee and ring doctor at upcoming Muay Thai competitions.

## Data Availability

Data and materials are available to other parties for research purposes after a data-sharing agreement plan is agreed to and signed. Those interested should contact the corresponding author. hhallaceli@mku.edu.tr

## Abbreviations

MMA: Mixed martial arts
IFMA: The International Federation of Muay Thai Associations
RSC: Referee Stops Contest
